# Inhaled carbon monoxide protects time-dependently from loss of hypoxic pulmonary vasoconstriction in endotoxemic mice

**DOI:** 10.1186/s12931-015-0274-7

**Published:** 2015-09-29

**Authors:** Nora Jahn, Regis R. Lamberts, Cornelius J. Busch, Maria T. Voelker, Thilo Busch, Marleen J. A. Koel-Simmelink, Charlotte E. Teunissen, Daniel D. Oswald, Stephan A. Loer, Udo X. Kaisers, Jörg Weimann

**Affiliations:** Department of Anaesthesiology and Intensive Care Medicine, University of Leipzig, Leipzig, Germany; Department of Anaesthesiology, Institute for Cardiovascular Research (ICaR-VU), VU University Medical Centre, Amsterdam, The Netherlands; Department of Anaesthesiology, Ruprecht-Karls-University, Heidelberg, Germany; Department of Clinical Chemistry, Neurological Laboratory and Biobank, VU University Medical Centre, Amsterdam, The Netherlands; Department of Anaesthesiology, Universitätsklinikum, Münster, Germany; Department of Anaesthesia and Intensive Care Medicine, Sankt Gertrauden-Krankenhaus, Berlin, Germany

**Keywords:** CO, HPV, Pulmonary circulation, Sepsis, Endotoxemia

## Abstract

**Background:**

Inhaled carbon monoxide (CO) appears to have beneficial effects on endotoxemia-induced impairment of hypoxic pulmonary vasoconstriction (HPV). This study aims to specify correct timing of CO application, it’s biochemical mechanisms and effects on inflammatory reactions.

**Methods:**

Mice (C57BL/6; *n* = 86) received lipopolysaccharide (LPS, 30 mg/kg) intraperitoneally and subsequently breathed 50 ppm CO continuously during defined intervals of 3, 6, 12 or 18 h. Two control groups received saline intraperitoneally and additionally either air or CO, and one control group received LPS but breathed air only. In an isolated lung perfusion model vasoconstrictor response to hypoxia (FiO_2_ = 0.01) was quantified by measurements of pulmonary artery pressure. Pulmonary capillary pressure was estimated by double occlusion technique. Further, inflammatory plasma cytokines and lung tissue mRNA of nitric-oxide-synthase-2 (NOS-2) and heme oxygenase-1 (HO-1) were measured.

**Results:**

HPV was impaired after LPS-challenge (*p* < 0.01). CO exposure restored HPV-responsiveness if administered continuously for full 18 h, for the first 6 h and if given in the interval between the 3^rd^ and 6^th^ hour after LPS-challenge (*p* < 0.05). Preserved HPV was attributable to recovered arterial resistance and associated with significant reduction in NOS-2 mRNA when compared to controls (*p* < 0.05). We found no effects on inflammatory plasma cytokines.

**Conclusion:**

Low-dose CO prevented LPS-induced impairment of HPV in a time-dependent manner, associated with a decreased NOS-2 expression.

## Background

Hypoxic pulmonary vasoconstriction (HPV) represents the main physiological mechanism to match ventilation to perfusion in the lungs and is crucial for appropriate systemic oxygenation [1, 2]. However, loss of HPV during inflammatory conditions, e.g. sepsis, may lead to intrapulmonary shunting of venous blood and severe hypoxemia [3–5]. The impairment of HPV represents an important pathophysiological factor during the development of sepsis-induced lung injury. Thus, the modification of HPV in acute lung injury might represent a potential therapeutic option to reduce its still high mortality of 30–50 % [6–9].

So far, the mechanism of impairment of HPV during sepsis remains elusive[10]. Experimental endotoxemia impairs HPV in several animal models, with increased production of cytokines and nitric oxide (NO) by the inducible nitric-oxide-synthase 2 (NOS-2) being responsible for loss of HPV [5, 11–15]. Preservation of HPV during experimental sepsis was achieved by administration of reactive oxygen species scavengers, by inhibition of NOS-2 with subsequent NO production or inhibition of the soluble guanylate cyclase as a molecular target of NO [5, 11, 15, 16].

Recently, carbon monoxide (CO) has been shown to be highly beneficial during acute lung injury due to its anti-inflammatory, anti-apoptotic and anti-proliferative properties [17–20]. Inhalation of low-dose CO reduced mortality and inflammatory response in various animal models of acute lung injury [17, 19, 21]. Moreover, recent clinical studies show a positive correlation between survival rates and endogenously produced CO levels in critically ill patients [22–24]. This marks CO as an intriguing therapeutic agent during acute inflammatory conditions, as it can easily be administered by inhalation [25–29]. In a previous study we could show that continuous inhalation of CO for more than 20 h improved HPV during murine endotoxemia in a dose dependent manner [30]. In the study presented we hypothesized that timing of CO inhalation is critical for sustaining HPV during endotoxemia and that the beneficial CO effects may be associated with a decrease in lung NOS-2 or HO-1 up-regulation or serum cytokine concentration.

## Material & Methods

All animal experiments were approved by the Governmental Animal Care Committee of the VU University medical centre, Amsterdam, the Netherlands and were conducted in conformity with “Guiding Principles in the Care and Use of Vertebrate Animals in Research and Training” and in accordance with the NIH guidelines for ethical animal research (*Guide for the Care and Use of Laboratory Animals*, NIH-publication No. 85–23, revised 1996). A total of 86 adult male mice with a body weight (bw) of 22.9 ± 0.2 g at the age of 8–12 weeks (C57BL/6; Harlan Laboratory, the Netherlands) were studied.

### Experimental groups

**Fig. 1:**
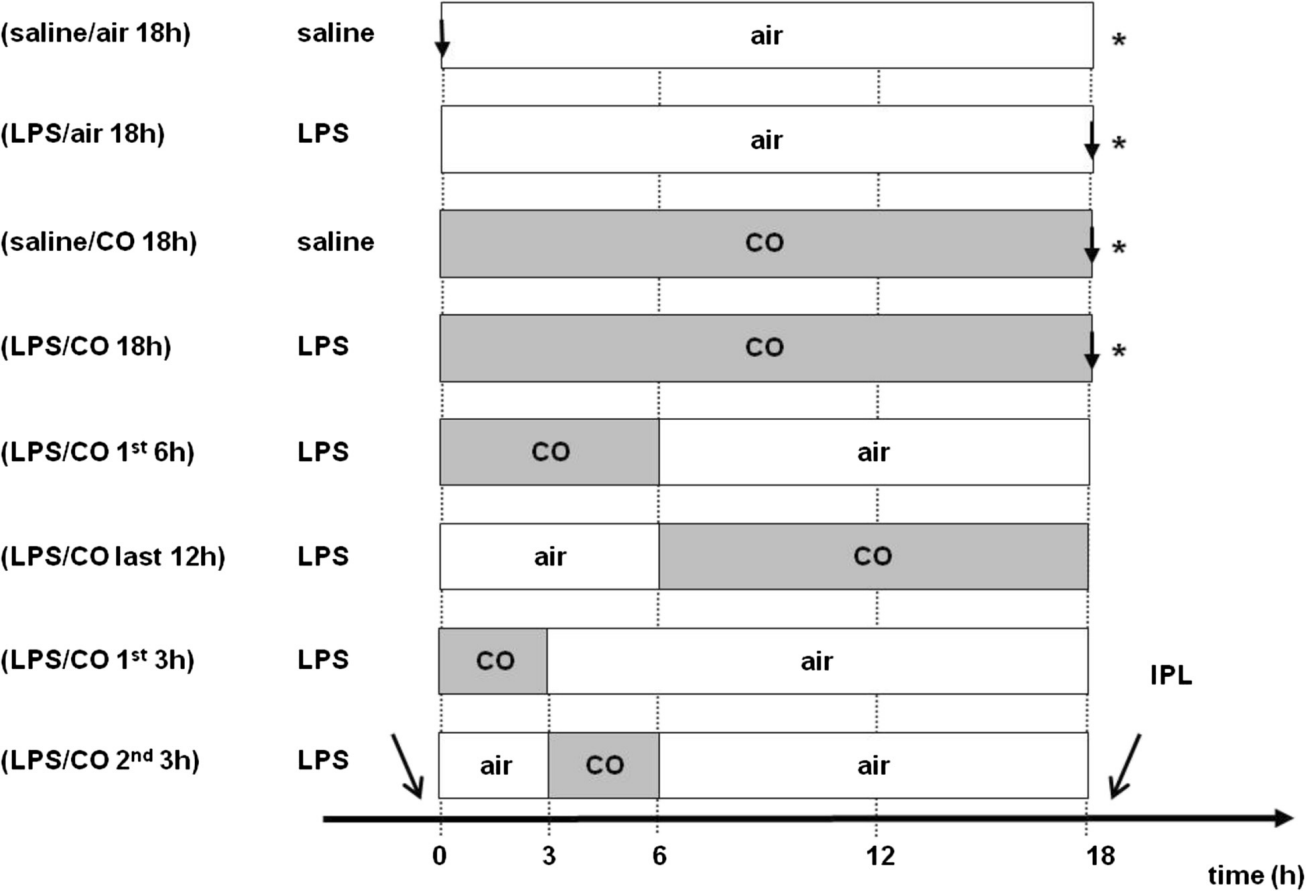
Experimental groups; Saline = controls were treated with saline i.p; LPS = mice were treated with 30 mg/kg bw Escherichia coli lipopolysaccharide i.p.; air (□) = mice were exposed to air; CO (■) = mice were exposed to 50 ppm CO; IPL = isolated perfused lung; ↓ = time points of tissue sampling in the molecular biology groups; * = collection of blood sample for cytokine detection; n = 8 per group

Mice were randomly assigned to eight groups of control and endotoxemic animals. For induction of endotoxemia, mice received an intraperitoneal (i.p.) injection of 30 mg/kg bw lipopolysaccharide (LPS; Escherichia coli 0111:B4 LPS, Sigma Aldrich Chemie GmbH, Steinheim, Germany) dissolved in normal saline. Controls received an equal amount of saline. After injection, animals were placed in a ventilated chamber and exposed to 50 ppm CO provided as a fixed gas mixture containing 50 ppm CO in 21 % oxygen and balanced nitrogen (Linde Gas Therapeutics Benelux B.V., Dieren, the Netherlands) either continuously for 18 h or during defined intervals for different groups. Controls were exposed to an adequate air mixture without CO. Configuration of experimental groups are given in Fig. 1.

### Isolated perfused mouse lung model

Mice (*n* = 70) received a lethal injection of pentobarbital sodium intraperitoneal (300 mg/kg bw, Ceva Sante Animale, Naaldwijk, the Netherlands) and were placed in a 37 °C water-jacketed chamber (Isolated Perfused Lung Size 1 type 839, Hugo- Sachs Elektronik, March-Hugstetten, Germany). After tracheotomy, volume-controlled ventilation (MiniVent type 845; Hugo-Sachs Elektronik, March-Hugstetten, Germany) was initiated with a tidal volume of 9 ml/kg bw, a positive end-expiratory pressure of 2 cm H_2_O and a respiratory rate of 100 breaths/min using a normoxic (FiO_2_ = 0.21) gas mixture (21 % O_2_, 5 % CO_2,_, balanced N_2_, Linde Gas Therapeutics Benelux B.V., Dieren, the Netherlands). After median sternotomy, the right ventricle was punctured for collection of a blood sample for cytokine measurements and subsequent injection of 10 U heparin, followed by exsanguination. For perfusion, cannulas (stainless steel, internal diameter 1 mm; Isolated Perfused Lung Size 1 type 839) were inserted into the pulmonary artery via the right ventricle and into the left atrium via the left ventricle. Lungs were perfused with a modified “Hanks’ balanced salt solution” (Merck, KGaA, Darmstadt, Germany) containing the following salts: 136.89 mmol/l NaCl, 5.37 mmol/l KCl, 0.34 mmol/l Na_2_HPO_4_, 0.44 mmol/l KH_2_PO_4_, 1.26 mmol/l CaCl_2_, 0.81 mmol/l MgSO_4_, 5.56 mmol/l D-Glucose, 4.17 mmol/l NaHCO_3_. Bovine serum albumin (3 %, Sigma-Aldrich Chemie GmbH, Steinheim, Germany) and dextran (3 %, Sigma-Aldrich Chemie GmbH, Steinheim, Germany) were added to the perfusate to prevent pulmonary edema [15, 31, 32]. Indomethacin (30 mmol/l, Sigma-Aldrich Chemie GmbH, Steinheim, Germany) and the non-selective nitric oxide synthase inhibitor L-NAME (1 mmol/l, Nω-Nitro-l-Argininmethylester; Sigma-Aldrich Chemie GmbH, Steinheim, Germany) were added to the perfusate to inhibit endogenous prostaglandin and NO synthesis, respectively [15, 31, 32]. Sodium bicarbonate (B. Braun Melsungen AG, Melsungen, Germany) was added to adjust the perfusate pH between 7.34–7.45. Perfusion was realized with a roller pump (ISM834A, IsmatecSA, Labortechnik-Analytik, Glattbrugg-Zürich, Switzerland) at 50 ml⋅kg^−1^⋅min^−1^ with a non-recirculating system at 37 °C, maintaining left atrial pressure (LAP) at 2 mmHg. Perfusate flow (Q) was adjusted using an in-line flow probe and flowmeter (T402, Transonic-Systems Inc., Ithaca, New York, USA). Pulmonary artery pressure (PAP) and LAP were measured via saline-filled pressure transducers (Medex Medical GmbH & Co KG, Klein-Winterheim, Germany) and a transbridge amplifier (TBM4M; World Precision Instruments, Berlin, Germany), and PAP, LAP, and Q were recorded at 150 Hz per channel on a personal computer using a data acquisition system (WinDaq™, DATAQ Instruments, Akron, OH, USA).

### Quantification of HPV

For quantification of HPV, we evaluated the hypoxia induced increase in pulmonary artery pressure, measured via a cannula inserted into the pulmonary artery [15, 31, 32]. Initially, lungs were ventilated with the normoxic gas mixture for 5 min and perfused at a constant flow rate of 50 ml⋅kg^−1^⋅min^−1^ (baseline perfusion), while maintaining a stable LAP value of 2 mmHg. Afterwards, lungs received a hypoxic gas mixture (FiO_2_ = 0.01; 1 % O_2_, 5 % CO_2_ and 94 % N_2;_ Linde Gas Therapeutics Benelux B.V., Dieren, the Netherlands). PAP was measured again after reaching a steady-state (approximately 10 to 15 min after initiation of hypoxic ventilation). HPV response was defined as increase of PAP in percent of baseline PAP during normoxic ventilation [15, 31, 32].

### Quantification of pulmonary capillary pressure

**Fig. 2:**
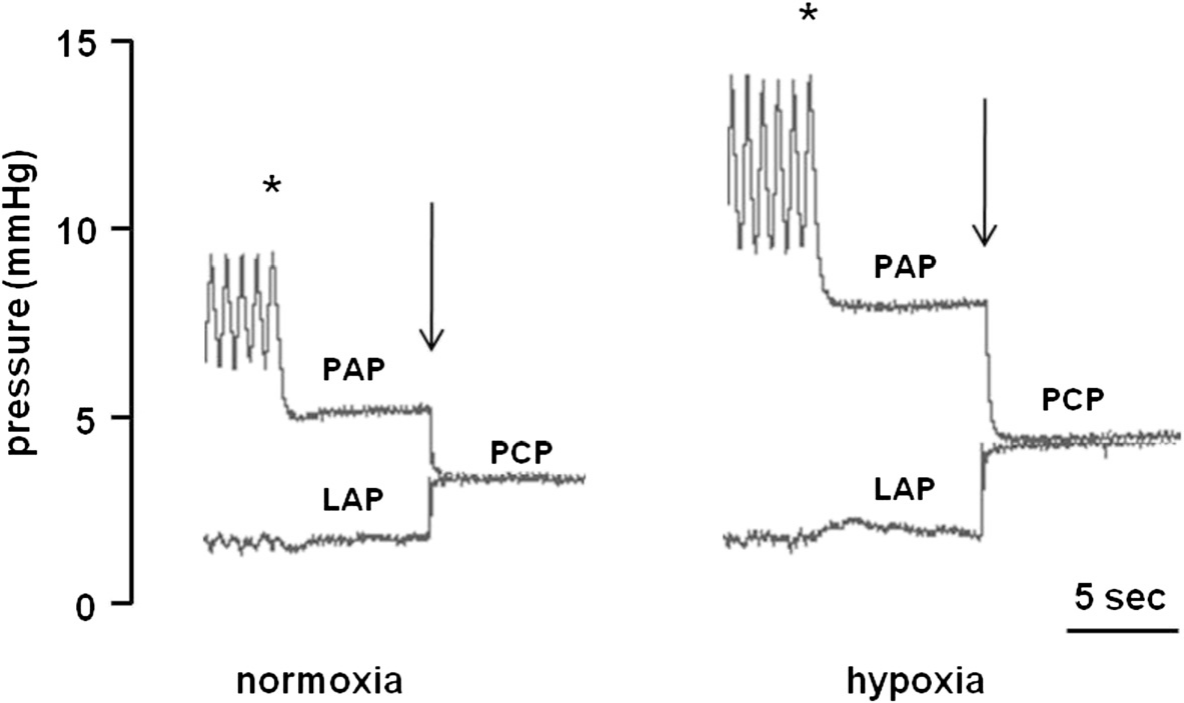
Double occlusion; Original recording of two successive double occlusion maneuvers (↓) during normoxic (21 % O_2_) and hypoxic (1 % O_2_) ventilation in an isolated perfused mouse lung. PAP = pulmonary artery pressure, LAP = left atrial pressure, PCP = pulmonary capillary pressure. Note that hypoxic ventilation predominantly causes arterial rather than venous vasoconstriction. *denotes interruption of ventilation

Pulmonary capillary pressure was estimated by the double occlusion technique (DBO) to evaluate the resistance in the pulmonary circulation [33, 34]. This technique is done as follows: after turning off the respirator in expiration, inflow and outflow through the lungs were stopped simultaneously for 5 s using two electromagnetic micro-valves (Clippard Minimatic, Cincinnati, OH). PAP and LAP were allowed to equilibrate and the resulting pressure represents the pressure in the pulmonary capillaries (PCP) [33] (see Fig. 2). During constant flow perfusion, total pulmonary vascular resistance is represented by the pressure drop across the pulmonary vasculature, expressed as the difference between PAP and LAP. Thus, the contribution of the pre-capillary, arterial part of pulmonary vasculature to total pulmonary vascular resistance can be expressed as PAP reduced by PCP and accordingly, the contribution of the post-capillary, venous part as PCP minus LAP. DBO values were included when a stable PCP curve was registered with complete equilibration of PAP and LAP (80 % inclusion).

We applied this technique during normoxic and hypoxic ventilation.

### Cytokine measurements

Serum cytokine concentrations of IL-1β, IL-2, IL-4, IL-5, IL-6, IL-10, IL-12, IL-13, TNFα, INFγ, GM-CSF and MCP-1 were measured in four randomly chosen animals from each the following groups: (saline/air 18 h), (LPS/air 18 h), (saline/CO 18 h) and (LPS/CO 18 h). Measurements were done using x-MAP (multi analyte profiling) technology with a pre mixed multiplex cytokine assay (Milliplex Mouse Cytokine Premixed 12 Plex Immunoassay Kit, Millipore B.V., Amsterdam, the Netherlands) and an automated Bio-plexTM 200 system (Cat# 171-000001FS1, Bio-Rad Laboratories B.V., Veenendaal, the Netherlands) with data-detection using the attendant software (Bio-plex Manager TM Software 4.1, Bio-Rad Laboratories B.V., Veenendaal, the Netherlands).

### Molecular biology

For determination of LPS- and CO-induced changes in mRNA expression, HO-1, HO-2 and NOS-2 were measured in the following groups: (saline/air18h), (LPS/air 18 h), (saline/CO 18 h) and (LPS/CO 18 h). In these animals, lungs were perfused with iced physiological saline for one minute at 50 ml⋅kg^−1^⋅min^−1^ flow (see isolated lung protocol for details) and subsequently harvested for further analysis. RNA was isolated from mouse lungs using Trizol reagent (Invitrogen Life Technologies, Carlsbad, CA), and complementary DNA was generated with iScript cDNA Synthesis Kit (BioRad Laboratories, Richmond, CA). Quantitative reverse-transcriptase PCR was performed with the ABI Prism 7000 Sequence Detection System (Applied Biosystems, Foster City, CA) using specific primers (for NOS-2 (TCTTTGACGCTCGGAACTGTAG, TGATGGCCGACCTGATGTT), HO-1 (GGGTGACAGAAGAGGCTAAG, GTGTCTGGGATGAGCTAGTG), HO-2 (ACCGAGCAGAAAATACCCAGT, GTTGCGGTCCATTTCCTCCTC), 18S (TCAAGAACGAAAGTCGGAGG, GGACAT CTAAGGGCATCAC)) and SYBR® Green PCR Master Mix (Applied Biosystems, Foster City, CA). Postamplification dissociation curves were performed to verify the presence of a single amplification product in the absence of DNA contamination. Changes in expression of the gene of interest were determined using the Ct-method with normalization to 18S ribosomal RNA.

### Statistical analysis

All values are expressed as means ± standard deviation (SD). To compare differences between experimental groups one-way or two-way ANOVA was performed where appropriate. When significant differences were detected, Bonferroni post hoc least significant difference test for planned comparisons was used. All calculation were performed with GraphPad Prism 5 (GraphPad Prism 5, La Jolla, CA).

## Results

### General response to LPS and CO

18 h after LPS-challenge mice showed lethargy, piloerection and diarrhoea with no visible CO effects on general appearance.

### Pulmonary vascular pressure during normoxia

**Fig. 3:**
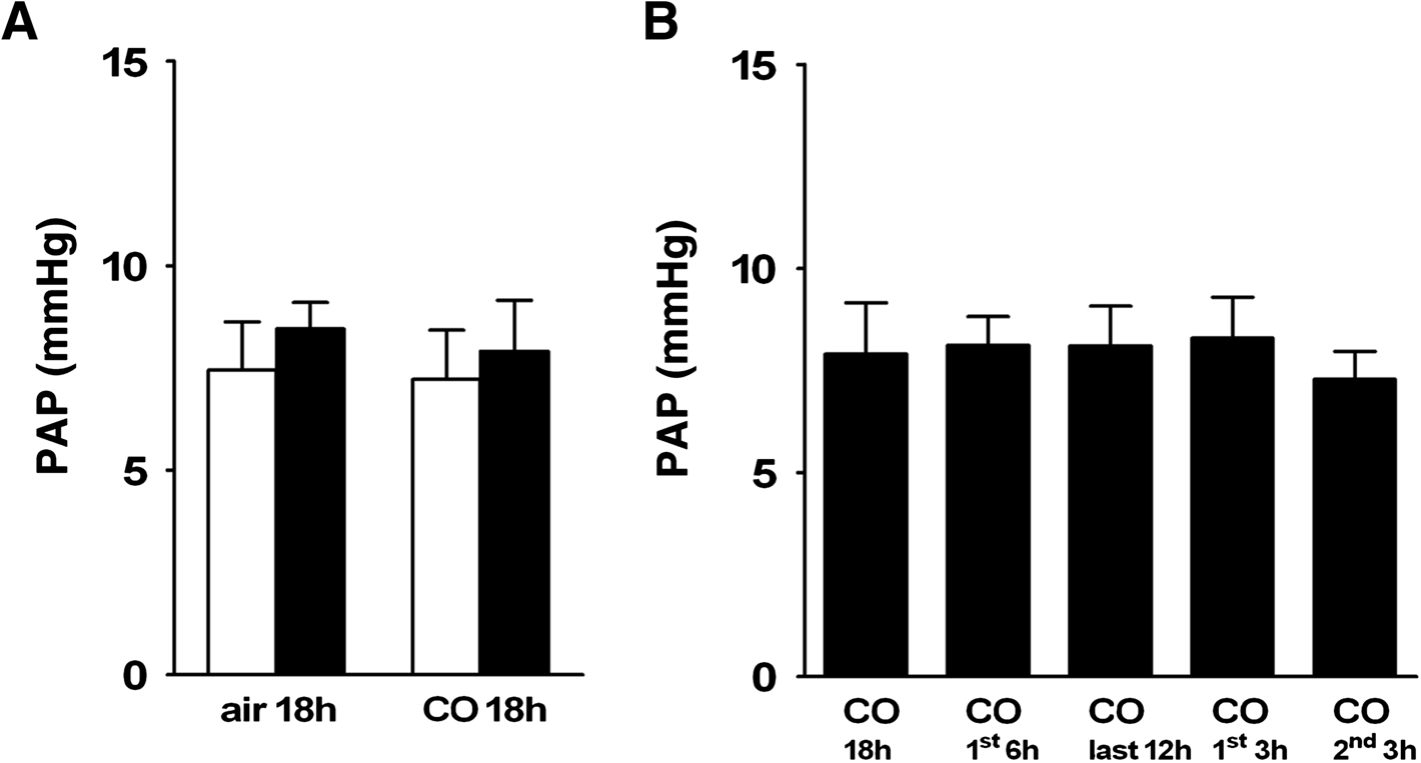
Baseline pulmonary artery pressure during normoxic ventilation; **a**. Average values of baseline pulmonary artery pressure (= PAP) during ventilation with 21 % O_2_ in lungs obtained from saline-treated controls (open bars) and from lipopolysaccharide-treated mice (LPS, black bars) after exposure to air or to 50 ppm CO for 18 h. Baseline PAP during nomoxic ventilation was not altered by LPS treatment or CO exposure. **b**. Average values of baseline PAP in lungs obtained from LPS-treated mice after exposure to 50 ppm CO for the first 6 h, the last 12 h, the first 3 h and the second 3 h after LPS challenge. Baseline PAP in LPS-treated animals was not affected by CO, independently of time and duration of CO exposure compared to CO exposure for 18 h. For better comparability values for the group (LPS/CO 18 h) are also depicted in figure B. Data are presented in mmHg as mean ± SD; *n* = 8 per group

**Fig. 4:**
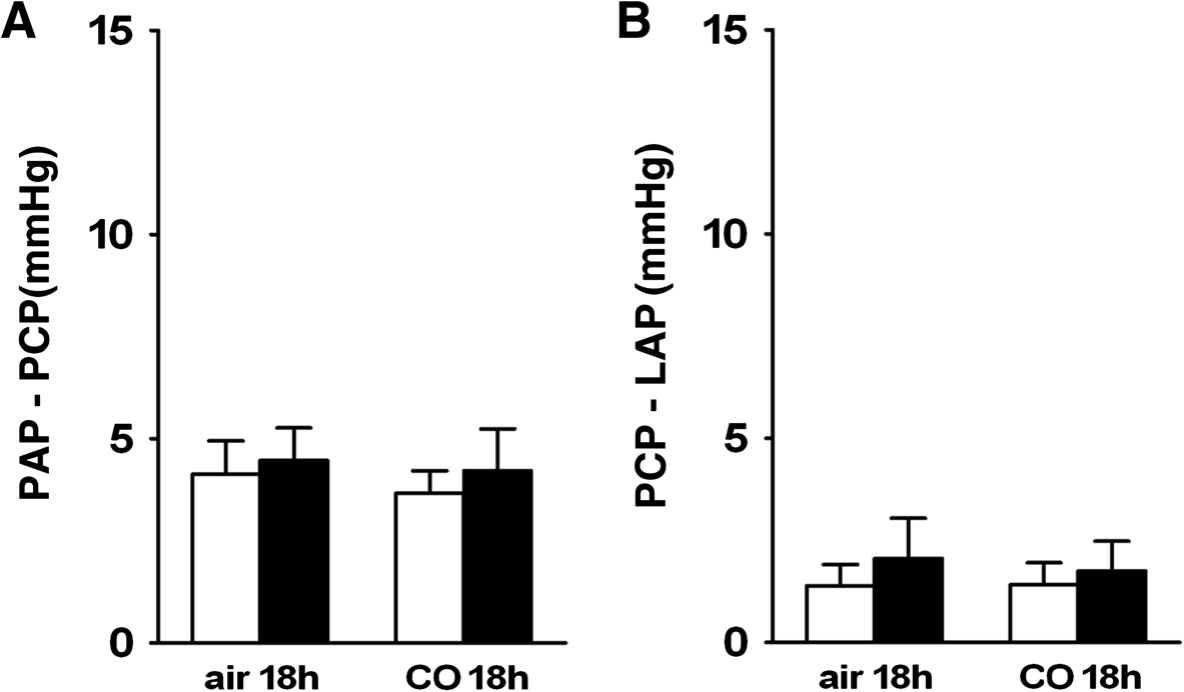
Arterial and venous fraction of pulmonary vascular resistance during normoxic ventilation; **a**. Average values of arterial resistance (PAP-PCP = pulmonary artery pressure - pulmonary capillary pressure) during normoxic ventilation (21%O_2_) in lungs obtained from saline-treated controls (open bars) and from LPS-treated mice (black bars) after exposure to air or to 50 ppm CO for 18 h. No CO- or LPS-induced alterations in arterial resistance during normoxic ventilation were found. **b**. Average values of venous resistance (PCP-LAP = pulmonary capillary pressure - left atrial pressure, with LAP = 2 mmHg) during normoxic ventilation in lungs obtained from saline-treated controls (open bars) and from LPS-treated mice (black bars) after exposure to air or to 50 ppm CO for 18 h. No CO- or LPS-induced alterations in venous resistance during normoxic ventilation were found. Data are presented in mmHg as mean ± SD; *n* = 6–8 per group

Baseline pulmonary artery pressure (PAP) during normoxic ventilation (FiO_2_ = 0.21) did not differ between LPS- and saline-treated animals. CO exposure for 18 h did not affect baseline PAP during normoxia in LPS- or saline-treated animals (see Fig. 3a). Baseline PAP was not altered by CO, independently of time and duration of CO exposure (see Fig. 3b). During baseline perfusion no significant LPS- or CO-induced changes in arterial or venous fraction of total pulmonary vascular resistance were detected (see Fig. 4a and b).

### Pulmonary vascular pressure during hypoxia

**Fig. 5:**
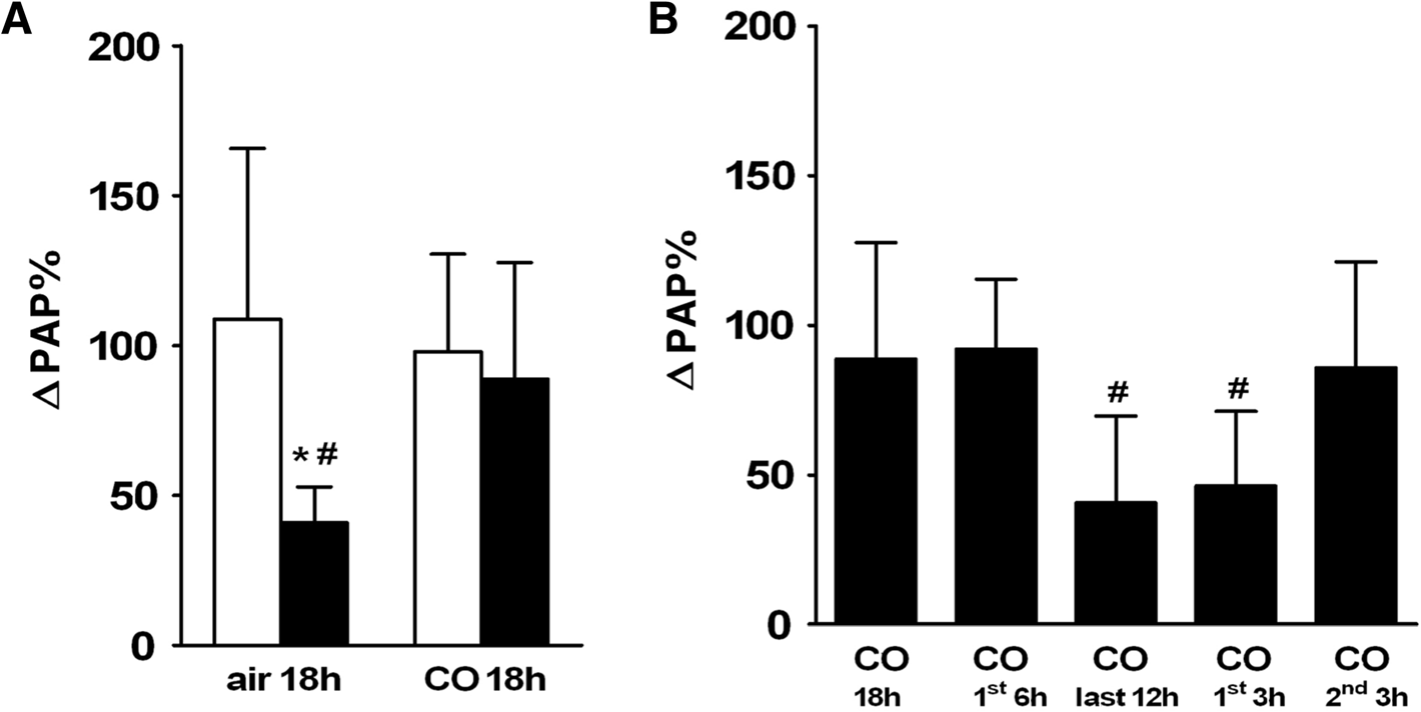
Hypoxic pulmonary vasoconstriction expressed as increase of pulmonary artery pressure during hypoxic ventilation; **a**. Average values of hypoxia induced vasoconstriction expressed as increase of pulmonary artery pressure (ΔPAP) in percent of baseline PAP in lungs obtained from saline-treated controls (open bars) and from LPS-treated mice (black bars) after exposure to air or to 50 ppm CO for 18 h. Hypoxia induced vasoconstriction was decreased in LPS-treated mice, which was prevented by exposure to 50 ppm CO for 18 h. **b**. Average values of ΔPAP in lungs obtained from LPS-treated mice after exposure to 50 ppm CO for the first 6 h, the last 12 h, the first 3 h and the second 3 h after LPS challenge. LPS-induced impairment of hypoxic pulmonary vasoconstriction was prevented by CO exposure for the first 6 h and the second 3 h after LPS challenge, but not by CO exposure for the first 3 h or the last 12 h. For better comparability values for the group (LPS/CO 18 h) are also depicted in figure B. Data are presented as mean ± SD; *n* = 8 per group; *p < 0.01 vs. (saline/air 18 h); # *p* < 0.05 vs. (LPS/CO 18 h)

**Fig. 6:**
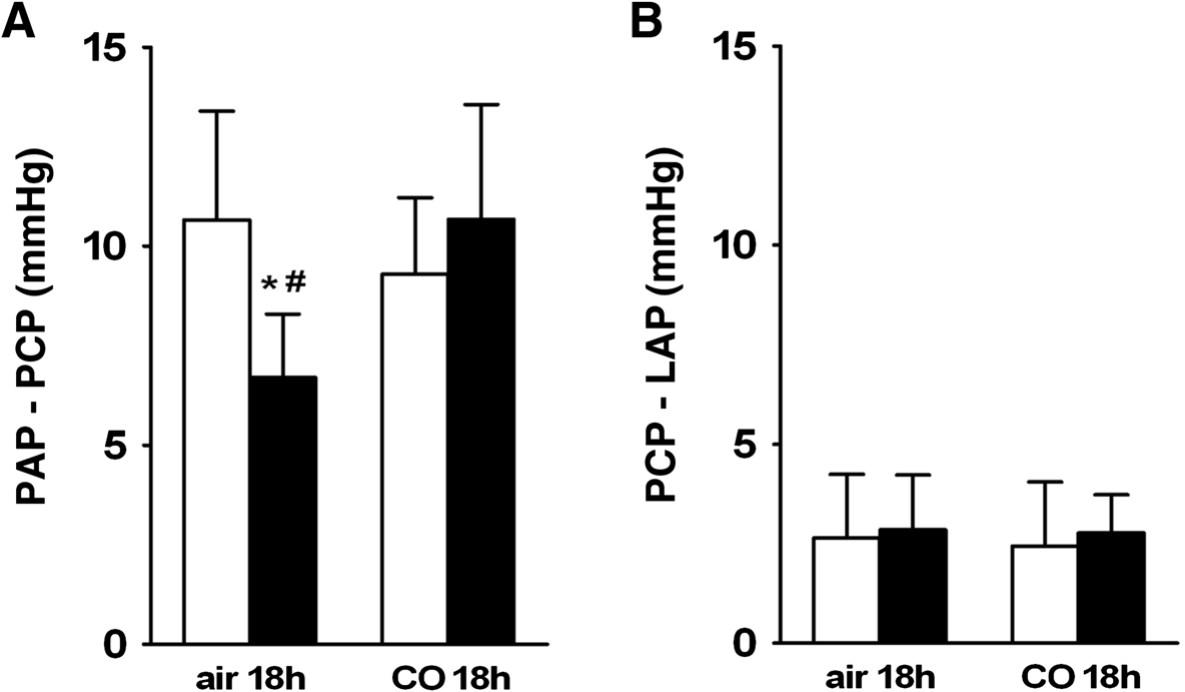
Pressure differences determining arterial and venous fractions of pulmonary vascular resistance during hypoxic ventilation; **a**. Pressure drop representing the arterial resistance (PAP-PCP = pulmonary artery pressure - pulmonary capillary pressure) during hypoxic ventilation (1%O_2_) in lungs obtained from saline-treated controls (open bars) and from LPS-treated mice (black bars) after exposure to air or to 50 ppm CO for 18 h. Arterial resistance during hypoxia was decreased in LPS-treated mice, which was prevented by exposure to 50 ppm CO for 18 h. **b**. Pressure drop representing the venous resistance (PCP-LAP = pulmonary capillary pressure - left atrial pressure, with LAP = 2 mmHg) during hypoxic ventilation in lungs obtained from saline-treated controls (open bars) and from LPS-treated mice (black bars) after exposure to air or to 50 ppm CO for 18 h. No CO- or LPS-induced alterations in venous resistance during hypoxic ventilation were found. Data are presented as mean ± SD; *n* = 6–8 per group; *p < 0.05 vs. (saline/air 18 h); # < 0.05 vs. (LPS/CO 18 h)

Hypoxic ventilation (FiO_2_ = 0.01) increased PAP in lungs of controls (saline/air 18 h) from 7.5 ± 1.2 to 15.2 ± 3.4 mmHg (*p* < 0.01). In LPS-treated animals PAP was significantly lower during hypoxic ventilation compared to saline-treated controls (*p* < 0.01). LPS-treatment (LPS/air 18 h) resulted in a significant reduction of HPV response (see Fig. 5a). HPV response was attributable to a concomitant increase in arterial fraction of total pulmonary vascular resistance in both saline- and LPS-treated animals, respectively. The LPS-induced impairment of HPV was attributable to a reduction of arterial fraction of total pulmonary vascular resistance (*p* < 0.05 versus controls) with no changes in venous fraction see Fig. 6a and b).

### Effect of CO exposure (18 h) on HPV responsiveness

While CO had no effect on HPV in saline-treated animals with a hypoxia-induced increase in PAP from 7.2 ± 1.2 to 14.1 ± 2.0 mmHg, LPS-treated animals exposed to CO for 18 h had a significantly higher HPV with a hypoxia-induced increase in PAP from 7.9 ± 1.2 to 14.7 ± 3.1 mmHg (*p* < 0.05 versus (LPS/air 18 h); see Fig. 5a). There was no detectable difference in HPV of controls (saline/air 18 h) and LPS-treated animals, exposed to CO for 18 h. Accordingly, the LPS-induced reduction in arterial fraction of total pulmonary vascular resistance during hypoxic ventilation, which accounted for the impairment of HPV, was not detectable in LPS-treated animals exposed to CO. LPS-treated animals exposed to CO for 18 h had a significantly higher arterial fraction of total pulmonary vascular resistance during hypoxic ventilation compared to LPS-treated animals without CO exposure (*p* < 0.05 vs. (LPS/air 18 h). Following CO exposure, the arterial fraction of total pulmonary vascular resistance in LPS-treated animals was similar to that observed in saline-treated animals with no CO dependent changes in venous fraction (see Fig. 6a and b).

### Time-dependence of CO exposure

CO exposure for the first 6 h after LPS injection (LPS/CO 1^st^ 6 h) followed by 12 h air exposure lead to a significant increase of HPV response, with a hypoxia-induced increase in PAP from 8.1 ± 0.7 to 15.5 ± 1.9 mmHg with no detectable difference in HPV between 18 h and 6 h CO exposure. Yet, CO exposure starting 6 h after LPS injection (LPS/CO last 12 h) resulted in a significantly lower HPV compared to 18 h CO exposure (*p* < 0.05 versus (LPS/CO 18 h); see Fig. 5b). Hereby, PAP increased from 8.1 ± 1.0 to 11.4 ± 2.4 mmHg during hypoxic ventilation. CO exposure for the first 3 h after LPS injection (LPS/CO 1^st^ 3 h) resulted in no improvement of HPV with a significantly lower ΔPAP compared to 18 h CO (*p* < 0.05) with a hypoxia-induced increase in PAP from 8.3 ± 1.0 to 12.2 ± 2.7 mmHg. However, CO exposure for the second 3 h after LPS injection (LPS/CO 2^nd^ 3 h) did lead to a significant elevation in HPV after LPS-challenge with a hypoxia-induced increase in PAP from 7.3 ± 0.7 to 13.4 ± 1.4 mmHg with no detectable difference in HPV between CO treatment for the entire 18 h period, the first 6 h or the second 3 h (see Fig. 5b).

### Cytokine serum concentrations

All measured serum cytokine levels were increased after LPS treatment. However, there was no detectable CO effect on serum cytokine concentrations in this two groups (LPS/air 18 h vs. LPS/CO 18 h) (see Table 1).

**Table 1:** Serum concentration of cytokines (in pg/ml) 18 h after LPS-challenge

Cytokines	(saline/air 18 h)	(LPS/air 18 h)	(saline/CO 18 h)	(LPS/CO 18 h)
IL-1β	6.7 ± 6.1^(3)^	923 ± 512	19.8 ± 8.6^(2)^	761 ± 302
IL-2	^(0)^	21.1 ± 7.6	^(0)^	24.7 ± 7.9
IL-4	0.9 ± 0.3^(2)^	9.4 ± 3.5	^(0)^	9.8 ± 4.0
IL-5	10.4^(1)^	415 ± 140	8.3 ± 5.9^(3)^	449 ± 300
IL-6	45.6 ± 4.0^(2)^	1,041,394 ± 1,305,421	5.2^(1)^	438,575 ± 120,555
IL-10	2.2 ^(1)^	1744 ± 1074	6.2 ± 5.7^(2)^	748 ± 281
IL-12	14.0 ± 9.2	1787 ± 485	33.1 ± 19.0^(2)^	1948 ± 383
IL-13	27.8 ± 7.2^(2)^	987 ± 518	30.3 ± 15.2	917 ± 281
TNF-α	^(0)^	269 ± 106	^(0)^	246 ± 64.8
INF-γ	^(0)^	3039 ± 1819	^(0)^	10,039 ± 10,856
GM-CSF	^(0)^	338 ± 77,9	32.8^(1)^	312 ± 50.7
MCP-1	17.4 ± 23.1^(2)^	122,499 ± 96,488	^(0)^	80,492 ± 29,767

Data are presented as mean ± SD; *n* = 4 per group; ^(n)^ = number of values within the detectable concentration range if n ≠ 4; ^(0)^ = all values were below the least detectable concentration

### Molecular biology

**Fig. 7:**
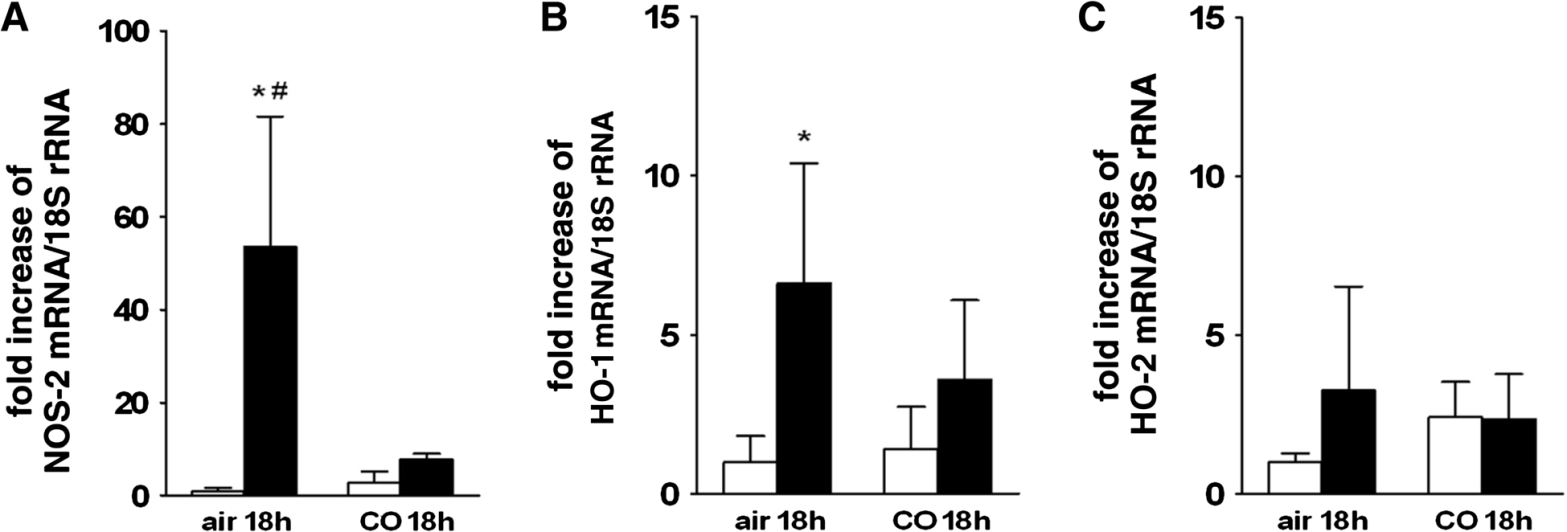
Increase of lung mRNA of NOS-2, HO-1 and HO-2 after LPS challenge; **a**. Average values of increase of NOS-2 mRNA in lungs from saline treated controls (open bars) and LPS-treated mice (black bars) after exposure to air or to 50 ppm CO for 18 h. NOS-2 mRNA was increased in LPS-treated mice more than 50fold compared to controls, which was prevented by exposure to 50 ppm CO for 18 h; *p < 0.001 vs. (saline/air 18 h); # *p* < 0.05 vs. (LPS/CO 18 h). **b**. Average values of increase of HO-1 mRNA in lungs from saline treated controls (open bars) and LPS-treated mice (black bars) after exposure to air or to 50 ppm CO for 18 h. HO-1 mRNA was increased in LPS-treated mice compared to controls, which was prevented by exposure to 50 ppm CO for 18 h; *p < 0.05 vs. (saline/air 18 h). **c**. Average values of increase of HO-2 mRNA in lungs from saline treated controls (open bars) and LPS-treated mice (black bars) after exposure to air or to 50 ppm CO for 18 h. HO-2 mRNA was not increased in LPS-treated mice compared to controls, which was not altered by exposure to 50 ppm CO for 18 h. Data are presented as mean ± SD

18 h after LPS injection and inhalation of air only, animals showed an increase of NOS-2 mRNA of 54-fold compared to saline controls (*p* < 0.001). Exposure to CO for 18 h did not affect NOS-2 expression in lungs of saline-treated mice. However, the tissue levels of NOS-2 mRNA in LPS treated mice having received CO was significantly reduced compared to LPS treated animals without CO exposure (*p* < 0.05; see Fig. 7a). HO-1 mRNA was significantly increased 7-fold in LPS treated animals 18 h after LPS-injection and inhalation of air (*p* < 0.05 versus controls). After exposure to 50 ppm CO for 18 h there was no significant difference in HO-1 mRNA concentration between LPS- and saline-treated animals. However, no significant difference between LPS treated animals exposed to air or to CO could be found either (see Fig. 7b). HO-2 mRNA expression was not influenced by LPS treatment or CO exposure (see Fig. 7c).

## Discussion

The main finding of the present study is that exposure to 50 ppm CO preserved hypoxic pulmonary vasoconstriction in endotoxemic mice, when administered during a short period in an early phase of endotoxemia. Preserved HPV was associated with recovered arterial resistance and accompanied by a reduction in lung NOS-2 mRNA expression.

We used an isolated perfused lung model to study the response of pulmonary vasculature to alveolar hypoxia in LPS-challenged mice. Administration of LPS induces endotoxemia, which impairs HPV in several animal models, including rodents, similar to loss of HPV during sepsis in critically ill patients [11, 12, 14, 15]. Therefore, LPS injection represents a highly reproducible and clinically relevant model of systemic inflammation with an impairment of HPV comparably to human sepsis. In combination with perfusion of isolated lungs it permits to study pulmonary vascular resistance under standardized conditions and via double occlusion it also provides information regarding the pulmonary microvasculature [5, 15, 31, 32]. The dose of 50 ppm CO used in our study was the most effective in prior dose–response experiments with continuous CO application for 21 h in a similar animal model by our group [30].

In the present study, we found a robust HPV in healthy control mice with 100 % increase of pulmonary vessel resistance during hypoxic ventilation (see Fig. 5a) similar to other studies [15, 31, 35–37]. Due to LPS-challenge, HPV was significantly diminished with a decreased pulmonary vessel resistance of 40 % comparably to results in other experimental models [15, 31, 37]. We here demonstrate for the first time, that HPV was preserved in isolated lungs of LPS-challenged mice after exposure to 50 ppm CO for 18 h resulting in a HPV-response of 90 %. HPV occurred due to an increase in arterial resistance (see Fig. 6a and b), which is consistent with previous studies using various animal models [36, 38, 39]. The hypoxia induced increase in arterial resistance was diminished after LPS-challenge, which was prevented by CO inhalation. Thus, CO-dependent preservation of HPV can be explained by preserved arterial function without increase in venous pressure or risk of pulmonary oedema.

The beneficial effects of continuously inhaled CO on pulmonary vascular tone during endotoxemia in our study are in line with previous studies, demonstrating beneficial effects of CO in models of mechanically, chemically or inflammatory induced acute lung injury [17, 19, 40]. Additionally, protective effects of CO on critically ill patients have become focus of intense clinical research [22–24]. In septic patients with acute lung injury, impairment of HPV leads to a decrease in arterial oxygen saturation, which might be a contributing factor to its high morbidity and mortality, underlining the clinical need for treatment to prevent loss of HPV during sepsis [1, 41, 42].

In a previous study, Mazzola et al. found an improved respiratory function in LPS challenged pigs, pre-treated with 250 ppm CO [14]. They reported ameliorations in cytokine expression, respiratory compliance, airway resistance and respiratory acidosis. Differently from our study, CO inhalation was stopped before LPS application and the protocol was terminated after 4 h. In addition, the animals received a continuous infusion of Ringer lactate to prevent extreme hypotension, which interfered with the blood pressure measurements. These factors may have contributed to the lack of evidence for a significant improvement in pulmonary hemodynamics in the investigation by Mazzola et al. [14]. In contrast, we were able to demonstrate a CO-induced preservation of HPV in endotoxemic lungs. As this is the main mechanism for ventilation perfusion matching, this might also have contributed to the reported improvement in respiratory function in the above mentioned study.

In our study, CO had no effect on baseline PAP in LPS- or saline-treated animals (see Fig. 3). This might seem conflicting with its direct vasodilating properties via activation of soluble guanylate cyclase (sGC) [28]. The 4.4-fold activation of sGC by CO, however, is considerably lower than the 130-fold activation by NO [43]. Accordingly, inhalation of 100 ppm exogenous CO did not affect PAP in healty mongrel dogs during hypoxia even after blockade nitric oxide synthase [44]. Attenuation of HPV by CO in isolated lungs of healthy pigs and rats was reported only at much higher inhaled concentrations in the range of 10 vol.% – equivalent to 100000 ppm [45, 46]. In addition it has to be noted that we did not apply CO in our study while ventilating the isolated perfused lungs.

Investigating the underlying molecular mechanism behind the beneficial CO effects on HPV during endotoxemia, we found no general anti-inflammatory effects of CO on elevated serum cytokine concentration after LPS-challenge in contrast to other studies [21, 47] (see Table 1). However, previously observed CO effects on cytokine concentration are inconsistent and furthermore appear to be dependent on the magnitude of the CO-concentration. Otterbein et al. report a concentration dependent decrease of pro-inflammatory serum cytokine concentration in LPS-challenged mice exposed to 10-500 ppm CO [27]. On the other hand, Ghosh et al. did not find a decrease in TNF-α concentration after 500 ppm CO exposure in LPS-challenged mice [12]. We used an even lower CO-concentration of 50 ppm, which may explain the absence of an effect on systemic cytokine concentration. Of interest, carboxy-Hb levels were measured in our prior dose–response experiments after more than 20 h of exposure to 50 ppm CO and varied between 4-9 % COHb, which is in line with previous studies [12, 17, 19, 30]. Thus, the observed preservation of HPV after CO exposure seems independent of a general anti-inflammatory effect of CO. However, it must be noted that a local anti-inflammatory effect of CO on cytokine expression in lung tissue cannot be excluded. Accordingly, Goebel et al. found no systemic anti-inflammatory effects with respect to cytokine expression after exposure to 250 ppm CO, but they reported a decrease in TNF-α and IL-1β mRNA expression in lung tissue of pigs with cardio-pulmonary bypass [18]. In the present study, cytokine expression in lung tissue was not determined and cytokine measurements were only performed at the endpoint of the experiment at 18 h after LPS injection. Therefore, CO dependent alterations in local cytokine expression in lung tissue or alterations in cytokine concentration during earlier time points of endotoxemia might also have contributed to preservation of HPV after CO exposure and may be an interesting focus of further studies on pulmonary inflammation.

In the present study, we also measured HO-1 and HO-2 mRNA as a marker of endogenous CO production and anti-inflammatory CO effects. According with previous findings, no changes in lung HO-2 mRNA, the constitutively expressed enzyme for endogenous CO production, were detected [48].

A change in HO-1 mRNA expression may be a sign of local anti-inflammatory CO effects during acute lung injury, which is supported by Clayton et al., who found an increase of lung HO-1 protein after hyperoxic injury in rats, which was reduced after CO exposure [49]. We found a 7-fold increase of lung HO-1 mRNA after LPS-challenge, confirming the up-regulation of HO-1 as part of the anti-inflammatory response mechanism [27, 29, 47]. Interestingly, in our data, we did not find the before mentioned significant reduction in HO-1 after CO inhalation in LPS treated animals. However, comparing controls and LPS-treated animals after CO exposure, the before mentioned difference in HO-1 expression was also lost (see Fig. 7b).

In our measurements of NOS-2 mRNA, we found that CO significantly reduced the LPS-induced 54-fold increase to an 8-fold increase, suggesting NOS-2 as an important molecular target of CO effects on HPV (see Fig. 7a). Therefore, our findings confirm the LPS-induced NOS-2 up-regulation as an important first step in endotoxemic impairment of HPV. It is important to note that the endogenous NO release at the time point of the measurement had no influence on the recorded HPV data. To specifically study pulmonary vasoconstrictor response to hypoxia at 18 h after LPS-injection we added indomethacine and L-NAME to the perfusate during isolated-perfused lung experiments. This approach allowed us to quantify HPV responsiveness independent of endogenous thromboxane and NO production as described before [15, 31, 32]. In a previous study we could show that impaired HPV is not due to counteracting endogenous NO production at 18 h after LPS-injection – i.e. cannot be restored by perfusing mouse lungs with L-NAME [15].

Our finding that the inhalation of 50 ppm CO reduced NOS-2 mRNA expression is in agreement with the results of other studies investigating the effects of similar CO doses. In IL-1beta stimulated hepatocytes from rats 250 ppm CO has been found to decrease NOS-2 protein and the formation of its active dimer [50]. In a rat model of LPS induced multiorgan failure 250 ppm CO prevented the up-regulation of iNOS and NO in lung tissue [51]. In a further investigation, 250 ppm CO inhibited LPS-induced activation of the transcription factor NF-kappa ß, a regulator of NOS-2 gene expression, in peritoneal mouse macrophages [52].

One of our central findings in search of the mechanism of protective CO effects on HPV was the identification of a critical time frame for inhaled CO to restore HPV after LPS challenge: CO exposure during the first 6 or the second 3 h after LPS injection preserved HPV in our study (HPV of 90 % or 86 %, respectively (see Fig. 5b)), whereas no improvement of HPV was observed when CO was administered during the last 12 h after LPS-challenge. Hence, CO had to be present at the beginning of endotoxemia in order to achieve a preserved HPV response.

Considering this precise time frame for beneficial actions of CO, the preservation of HPV may be achieved by inhibition of the pulmonary NOS-2 up-regulation and a consequent reduction in pulmonary NO production during early endotoxemia as NOS-2 deficient mice are protected from loss of HPV during sepsis [15]. A peak concentration of NOS-2 mRNA at 6 h following LPS challenge in mice with a subsequent increase in NOS-2 activity at 12 h which was reported by Salimuddin et al. is in agreement with the time interval during which inhaled CO restored HPV in our study [53]. Furthermore, previous studies demonstrated that inhibition of NOS-2 and dependent NO production must occur during an early phase of endotoxemia to preserve HPV, whereas inhibition of NO production at a later time point does not restore HPV [5, 15, 32]. Thus, Ullrich et al. found a sustained HPV in LPS-challenged mice, when NOS-2 dependent NO production was inhibited three hours after LPS-injection, which was not established by NOS-2 inhibition during measurement of HPV at 22 h after LPS-challenge [5]. Of note, the LPS-induced peak of NOS-2 expression around six hours after LPS-challenge reported in various investigations coincides with the period during which CO had to be administered for preservation of HPV in our study [4, 13, 15, 54].

Taken together, our results are in line with previous findings suggesting a) that an early LPS induced NOS-2 activity is responsible for an initial impairment of HPV and b) that HPV is subsequently maintained by other factors which have likely been produced or activated depending on the increased NO concentrations. Previous studies indicate a possible involvement of arginase which is coinduced by LPS with NOS-2 in lung tissue of mice and has been demonstrated to reach its concentration peak at 24 h after LPS application [53]. Induction of NOS-2 in murine macrophages has been found to increase arginase activity by S-nitrosylation of cysytein residues [55]. Furthermore, application of an aginase inhibitor 22 h after LPS challenge has actually been reported to restore HPV in mice [56].

In conclusion, exposure to low-dose CO in a concentration of 50 ppm prevents impairment of HPV in LPS-challenged mice. Preservation of HPV was due to sustained arterial function and independent of systemic serum cytokine concentration. Interestingly, CO had to be administered during the second 3 h of endotoxemia to bear its protective properties, indicating a limited time frame for protective CO effects. Preserved HPV was associated with a reduction in NOS-2 mRNA after LPS-challenge, suggesting an important key role of CO in prevention of inflammatory up-regulation of NOS-2 and consequent NO production. Our data implicate that application of low-dose CO might be considered as a future option for the treatment of inflammatory lung failure and the consequent hypoxemia.
